# Genome-wide identification and expression analysis of the SET domain-containing gene family in potato (*Solanum tuberosum* L.)

**DOI:** 10.1186/s12864-024-10367-2

**Published:** 2024-05-03

**Authors:** Vithusan Suppiyar, Venkata Suresh Bonthala, Asis Shrestha, Stephanie Krey, Benjamin Stich

**Affiliations:** 1 Institute for Quantitative Genetics and Genomics of Plants, Heinrich Heine University, Düsseldorf, 40225 Germany; 2 Cluster of Excellence On Plant Sciences, From Complex Traits Towards Synthetic Modules, Heinrich Heine University, Düsseldorf, 40225 Germany; 3 Present Address: Julius Kühn-Institut (JKI), Institute for Breeding Research On Agricultural Crops, Rudolf-Schick-Platz 3a, OT Groß Lüsewitz, Sanitz, 18190 Germany

**Keywords:** *Solanum tuberosum*, SET domain-containing genes, Histone lysine methylation, Abiotic stress, Pollen-specific expression, Epigenetics

## Abstract

Genes containing the SET domain can catalyse histone lysine methylation, which in turn has the potential to cause changes to chromatin structure and regulation of the transcription of genes involved in diverse physiological and developmental processes. However, the functions of SET domain-containing (StSET) genes in potato still need to be studied. The objectives of our study can be summarized as in silico analysis to (i) identify StSET genes in the potato genome, (ii) systematically analyse gene structure, chromosomal distribution, gene duplication events, promoter sequences, and protein domains, (iii) perform phylogenetic analyses, (iv) compare the SET domain-containing genes of potato with other plant species with respect to protein domains and orthologous relationships, (v) analyse tissue-specific expression, and (vi) study the expression of StSET genes in response to drought and heat stresses. In this study, we identified 57 StSET genes in the potato genome, and the genes were physically mapped onto eleven chromosomes. The phylogenetic analysis grouped these StSET genes into six clades. We found that tandem duplication through sub-functionalisation has contributed only marginally to the expansion of the StSET gene family. The protein domain TDBD (PFAM ID: PF16135) was detected in StSET genes of potato while it was absent in all other previously studied species. This study described three pollen-specific StSET genes in the potato genome. Expression analysis of four StSET genes under heat and drought in three potato clones revealed that these genes might have non-overlapping roles under different abiotic stress conditions and durations. The present study provides a comprehensive analysis of StSET genes in potatoes, and it serves as a basis for further functional characterisation of StSET genes towards understanding their underpinning biological mechanisms in conferring stress tolerance.

**Supplementary Information:**

The online version contains supplementary material available at 10.1186/s12864-024-10367-2.

## Introduction

The nucleosome, the fundamental unit of eukaryotic chromatin material, consists of two DNA strands wrapped around an octamer of histone proteins, which comprises two copies of each H2A, H2B, H3, and H4 protein [1]. Post-translational modifications, such as acetylation, methylation, phosphorylation, ubiquitination, and SUMOylation, covalently modify the N-terminal region of core histones [2, 3]. These modifications impact chromatin structure and accessibility and thereby can regulate gene expression [4, 5]. In plants, histone methylation is among the most well-understood histone modifications. This modification plays a crucial regulatory role in plant growth and development, reproductive processes, and response to environmental factors [5–8].

The SET domain-containing proteins, which share a highly conserved SET domain, mainly involved in catalysing histone lysine methylation [9], were first discovered in Suppressor of variegation 3–9 (Su(var)3–9), Enhancer of zeste (E(z)) and Trithorax (Trx) proteins in *Drosophila melanogaster* [10]. SET domain-containing proteins are involved in the methylation of lysine (K) residues of histones, such as H3 (K4, K9, K27, and K36) and H4 (K20) [11]. Typically, di-/tri-methylation of H3K4 and H3K36 can result in transcriptional inactivation, di-methylation of H3K9 and tri-methylation of H3K27 may promote gene silencing in both plants and animals [6, 12].

The SET domain is approximately 130 amino acids in length and comprises two non-contiguous regions: SET-N and SET-C, located at the N- and C-terminals of the primary structure, and an insert region known as SET-I [13] In plants, the SET domain-containing genes are reported to be involved in genomic alterations in addition to histone lysine methylation, e.g., intron retention [14] and DNA transposition [15]. Furthermore, SET domain-containing genes have also been associated with abiotic stress responses [16, 17], flowering time regulation [18], shoot branching [19], and carotenoid biosynthesis [20].

The SET domain-containing genes have been identified and functionally characterised for their roles in growth, development, and stress responses in several plant species, including *Arabidopsis thaliana* [21], *Camellia sinensis* [22], *Gossypium raimondii* [23]*, Malus domestica* [24]*, Oryza sativa* [25], *Populus trichocarpa* [26], *Setaria italica* [27], *Solanum lycopersicum* [28], and *Triticum aestivum* [29]. These studies comprehensively characterised the SET domain-containing genes, including the inference of phylogenetic relationships, investigation of the role of gene duplications on the expansion of this gene family, protein domain organisation, tissue-specific expression analysis and expression responses upon abiotic stresses. The phylogenetic analysis of SET domain-containing genes in the above-mentioned plant species displayed variations in clades ranging from 5 to 7. Li et al. [24] found that the specific protein domain composition contributes to the multiple functions of SET domain-containing genes in *Malus domestica*. In addition, they found that a recent genome-wide duplication event in *Malus domestica* mainly causes the expansion of this gene family. Yadav et al. [27] found differential expression of SiSET genes in *Setaria italica* during the late abiotic stress and hormonal treatments phase. However, no such study has been performed to identify and comprehensively analyse potato’s SET domain-containing gene family.

Potato, the most important non-cereal food crop, is a highly heterozygous autotetraploid species [30]. It holds the third rank in food production, following wheat and rice, and has an annual global production of over 376 million tons [31]. Potato suffer from various abiotic stresses due to climate change [32]. However, the functions of the SET domain-containing gene family in abiotic stresses in potatoes still need to be studied.

Therefore, the objectives of our study were to (i) identify the SET domain-containing genes in the potato genome, (ii) systematically analyse gene structure, chromosomal distribution, gene duplication events, promoter sequences, and protein domains, (iii) perform phylogenetic analyses, (iv) compare the SET domain-containing genes of potato with other plant species with respect to protein domains and orthologous relationships, (v) analyse tissue-specific expression, and (vi) study the expression of the SET domain-containing genes in response to drought and heat stresses.

## Results

### Genome-wide identification and analysis of StSET genes in potato

**Fig. 1:**
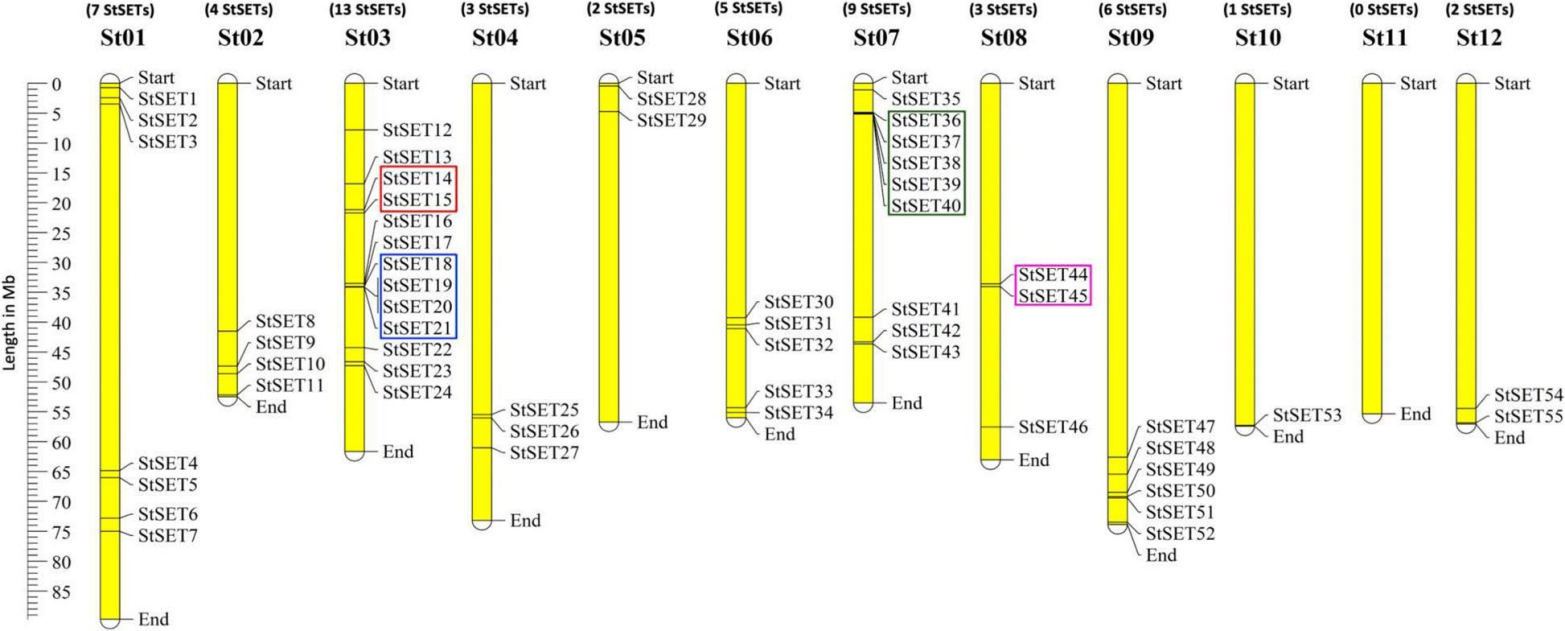
Physical mapping of StSET genes in the potato genome. The twelve potato chromosomes numbered from St01 – St12, and the number of StSET genes identified in respective chromosomes mentioned above the chromosome. StSET genes are numbered consecutively based on their position on the chromosomes (StSET01—StSET55). We excluded StSET56 and StSET57 genes from the physical mapping due to their mapping to sequences of unknown chromosomal locations. The scale bar on the left shows the chromosome length in Megabases (Mb). The tandemly duplicated gene clusters (TDG1—TDG4) of StSET are marked by different colour boxes. We visualised the physical mapping of StSET genes using MapChart v2.32 [33]

We obtained 51 genes with the BLASTP search and 73 genes with the HMMER search. Finally, we combined the HMMER and BLASTP search results to get 81 unique genes. After filtering the false positive genes by InterProScan and Pfam analyses, we obtained 57 SET domain-containing (PF00856) genes (StSETs) in the potato genome. However, we did not find additional genes containing a SET domain from the second BLASTP search of 57 StSET genes against the reference proteome sequences. We assigned a consecutive numbering to these genes based on their position on the chromosomes. The genes appeared on all chromosomes except chromosome 11. However, two genes (StSET56 and StSET57) mapped to sequences of unknown chromosomal locations. Chromosome 3 contained the highest number of StSET genes (13), followed by chromosome 7 (9), while chromosome 10 contained a single StSET gene (Fig. 1; Table S1).

**Fig. 2:**
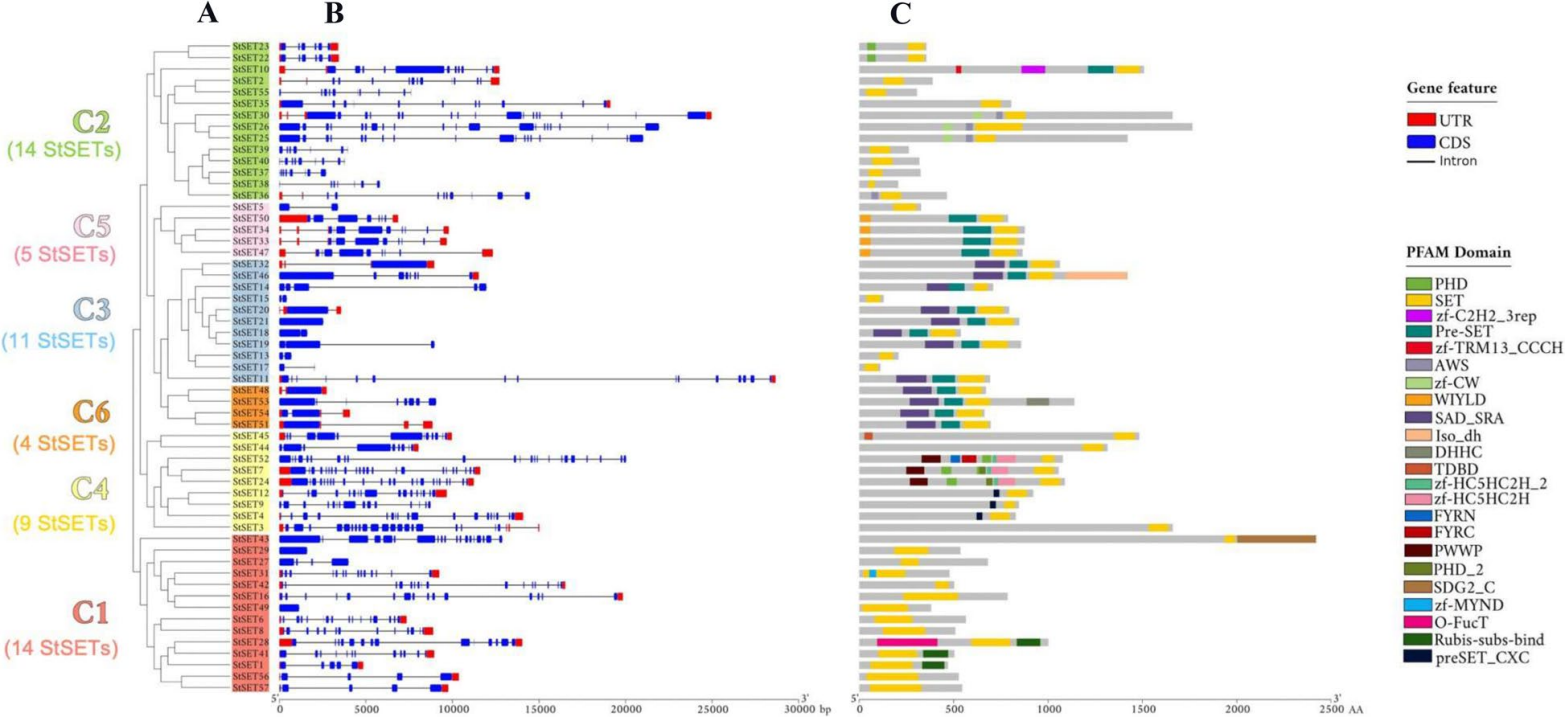
Gene structure and protein domain organisation of StSET genes with respect to their phylogenetic order. A). The estimated phylogenetic tree for StSET genes, B). Gene structure of StSET genes, and C). Protein domain organisation of StSET proteins. We visualised the phylogenetic tree, the gene structure and the protein domain organisations using TBTools v1.098696 [34]

The length of StSET gene sequences ranged from 430 to 28,651 nucleotides. Three genes, namely StSET21, StSET29, and StSET49, contained a single exon, while the remaining genes contained up to 24 exons (Fig. 2B; Table S1). The length of protein sequences of StSET genes ranged from 112 to 2421 amino acids. The proteins of StSET genes had an average and median molecular weight of 87.7 and 78.2 kilodaltons (kDa), respectively. The protein of StSET43 had the highest molecular weight of 276.5 kDa, while the protein of the StSET17 gene had the lowest molecular weight of 13 kDa. The StSET proteins had a theoretical pI spectrum of 4.51 to 9.47. We predicted that about 84% of the StSET proteins (48 StSETs) are unstable. Amino acid composition analysis showed that Serine (Ser), Glycine (Gly), Leucine (Leu), and Lysine (Lys) are the predominant amino acid residues of StSET proteins. The grand average of hydropathicity (GRAVY) values indicated that StSET proteins are hydrophilic (Table S2).

We found 23 unique protein domains in the protein sequences of StSET genes, including the SET domain (Fig. 2C; Table 1). About 38% of protein sequences of StSET genes (22 genes) contained only the SET domain, while the remaining genes contained diverse combinations of multiple protein domains along with the SET domain. For example, about 17% of protein sequences of StSET genes contained the combination of the SET, Pre_SET, and SAD_SRA protein domains, while one contained a combination of eight protein domains, such as SET, PWWP, FYRN, FYRC, PHD, PHD_2, zf-HC5HC2H_2, and zf-HC5HC2H (Table 2).

**Table 1:** List of protein domains identified in SET domain-containing genes across *Solanum tuberosum, Solanum lycopersicum, Arabidopsis thaliana,* and *Oryza sativa*. ✓indicates presence of a specific protein domain, while X indicates absence of a specific protein domain in the respective plant species

S. No	Pfam ID	Protein domain name	*Solanum tuberosum*	*Solanum lycopersicum*	*Arabidopsis thaliana*	*Oryza sativa*
1	PF02178	AT_hook	X	X	X	✓
2	PF17907	AWS	✓	✓	✓	✓
3	PS50216	DHHC	✓	X	X	X
4	PF14291	DUF4371	X	✓	X	X
5	PS51543	FYRC	✓	X	✓	✓
6	PS51542	FYRN	✓	X	✓	✓
7	PF14237	GYF_2	X	X	✓	X
8	PF00180	Iso_dh	✓	X	X	X
9	PF10250	O-FucT	✓	X	X	X
10	PF00628	PHD	✓	✓	✓	✓
11	PF13831	PHD_2	✓	✓	✓	✓
12	PF05033	Pre-SET	✓	✓	✓	✓
13	PF18264	preSET_CXC	✓	✓	✓	✓
14	PF00855	PWWP	✓	✓	✓	✓
15	PF09273	Rubis-subs-bind	✓	✓	✓	✓
16	PF02182	SAD_SRA	✓	✓	✓	✓
17	PF19633	SDG2_C	✓	✓	✓	✓
18	PF00856	SET	✓	✓	✓	✓
19	PF16135	TDBD	✓	X	X	X
20	PF10440	WIYLD	✓	✓	✓	✓
21	PF18868	zf-C2H2_3rep	✓	✓	✓	X
22	cd20146	zf-CW	✓	✓	✓	✓
23	PF13771	zf-HC5HC2H	✓	✓	✓	✓
24	PF13832	zf-HC5HC2H_2	✓	✓	✓	✓
25	PF15801	zf-MYND	✓	✓	✓	✓
26	PF11722	zf-TRM13_CCCH	✓	✓	X	X

**Table 2:** The number of SET genes in which a unique combination of protein domains identified in SET domain-containing genes is observed for *Solanum tuberosum, Solanum lycopersicum, Arabidopsis thaliana,* and *Oryza sativa*

S. No	Protein domain combinations	*Solanum tuberosum*	*Solanum lycopersicum*	*Arabidopsis thaliana*	*Oryza sativa*
1	SET	22	12	12	9
2	SET, Pre-SET, SAD_SRA	10	9	9	9
3	SET, Pre-SET, WIYLD	4	3	3	1
4	SET, preSET_CXC	3	2	3	2
5	SET, zf-CW, AWS	3	2	1	1
6	SET, Rubis-subs-bind	2	1	6	6
7	SET, PHD	2	2	2	2
8	SET, PWWP, PHD, PHD_2, zf-HC5HC2H_2, zf-HC5HC2H	2	2	3	0
9	SET, PWWP, FYRN, FYRC, PHD, PHD_2, zf-HC5HC2H_2, zf-HC5HC2H	1	0	0	1
10	SET, zf-TRM13_CCCH, zf-C2H2_3rep, Pre-SET	1	1	0	0
11	SET, Pre-SET, SAD_SRA, Iso_dh	1	0	0	0
12	SET, SDG2_C	1	1	0	1
13	SET, AWS	1	2	1	1
14	SET, TDBD	1	0	0	0
15	SET, zf-MYND	1	2	2	2
16	SET, Pre-SET, SAD_SRA, DHHC	1	0	0	0
17	SET, Rubis-subs-bind, O-FucT	1	0	0	0
18	SET, zf-HC5HC2H_2, zf-HC5HC2H, FYRN, FYRC, PWWP, PHD_2	0	0	2	0
19	SET, SAD_SRA	0	0	1	0
20	SET, zf-C2H2_3rep, Pre-SET	0	0	1	0
21	SET, SDG2_C, GYF_2	0	0	1	0
22	SET, DUF4371	0	1	0	0
23	SET, zf-HC5HC2H_2, zf-HC5HC2H, PHD_2, PHD	0	1	0	0
24	SET, PWWP, PHD_2, zf-HC5HC2H_2, zf-HC5HC2H	0	0	0	2
25	SET, Pre-SET, SAD_SRA, AT_hook	0	0	0	1
26	SET, Pre-SET, PHD	0	0	0	1
27	SET, zf-HC5HC2H_2, zf-HC5HC2H, PHD_2	0	0	0	1
28	SET, Pre-SET	0	0	0	1

The gene ontology (GO) enrichment analysis identified significantly enriched GO terms (*p* < 0.05) involved in various biological processes (56 GO terms), molecular functions (57 GO terms), and cellular components (55 GO terms). For example, 100% and about 82.5% of StSET genes were predicted to be involved in catalytic activity and response to stimulus, respectively (Figure S2; Table S3).

We predicted for approximately 93% of StSET genes a localisation in the nucleus, while for the others a localisation in the mitochondria (StSET1) or the chloroplast (StSET8 and StSET41) (Table S1) was predicted. Three genes (StSET28, StSET45, and StSET53) were predicted to have transmembrane helices (Table S1).

### Identification of duplicated StSET genes

We found four tandemly duplicated gene (TDG) clusters in StSET genes with cluster sizes from 2—5 genes. The TDG clusters contained about 23% of StSET genes. We found two TDG clusters with StSET genes on chromosome 3, while one was on chromosomes 7 and 8 (Fig. 1). We estimated the non-synonymous (Ka) and synonymous (Ks) substitution ratios (Ka/Ks) for each pair of tandemly duplicated StSET genes, and the ratios ranged from 0.39—0.99.

### Phylogenetic analysis of StSET genes

**Fig. 3:**
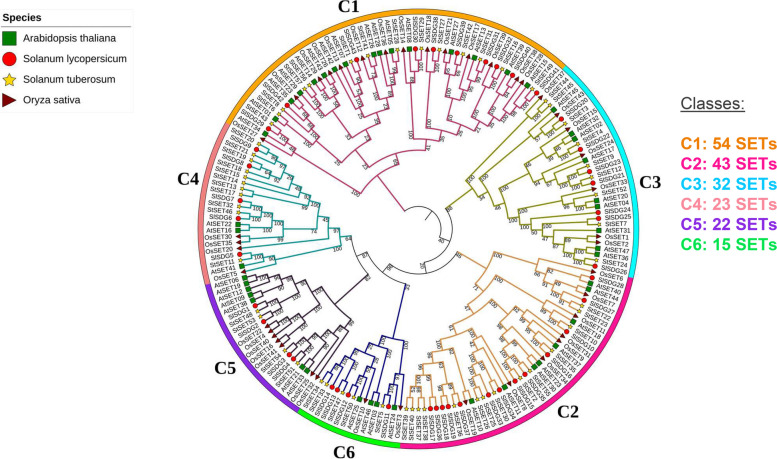
Phylogeny of SET domain-containing genes of potato and *Arabidopsis thaliana*, *Solanum lycopersicum*, and *Oryza sativa*. We visualised the computed phylogenetic trees using iTol [35]

We estimated a phylogenetic tree that clustered all StSET genes into six clades denoted as C1—C6 (Fig. 2A). The largest clades, C1 and C2, contained an equal number of StSET genes (14 genes in each clade), while the smallest clade (C6) contained four StSET genes. Further, we estimated a phylogenetic tree for SET domain-containing genes from *Solanum tuberosum*, *Solanum lycopersicum*, *Oryza sativa*, *and Arabidopsis thaliana*, and this phylogenetic tree also clustered all the genes into six clades denoted as C1—C6 (Fig. 3).

### Identification of cis-elements and conserved motifs

**Fig. 4:**
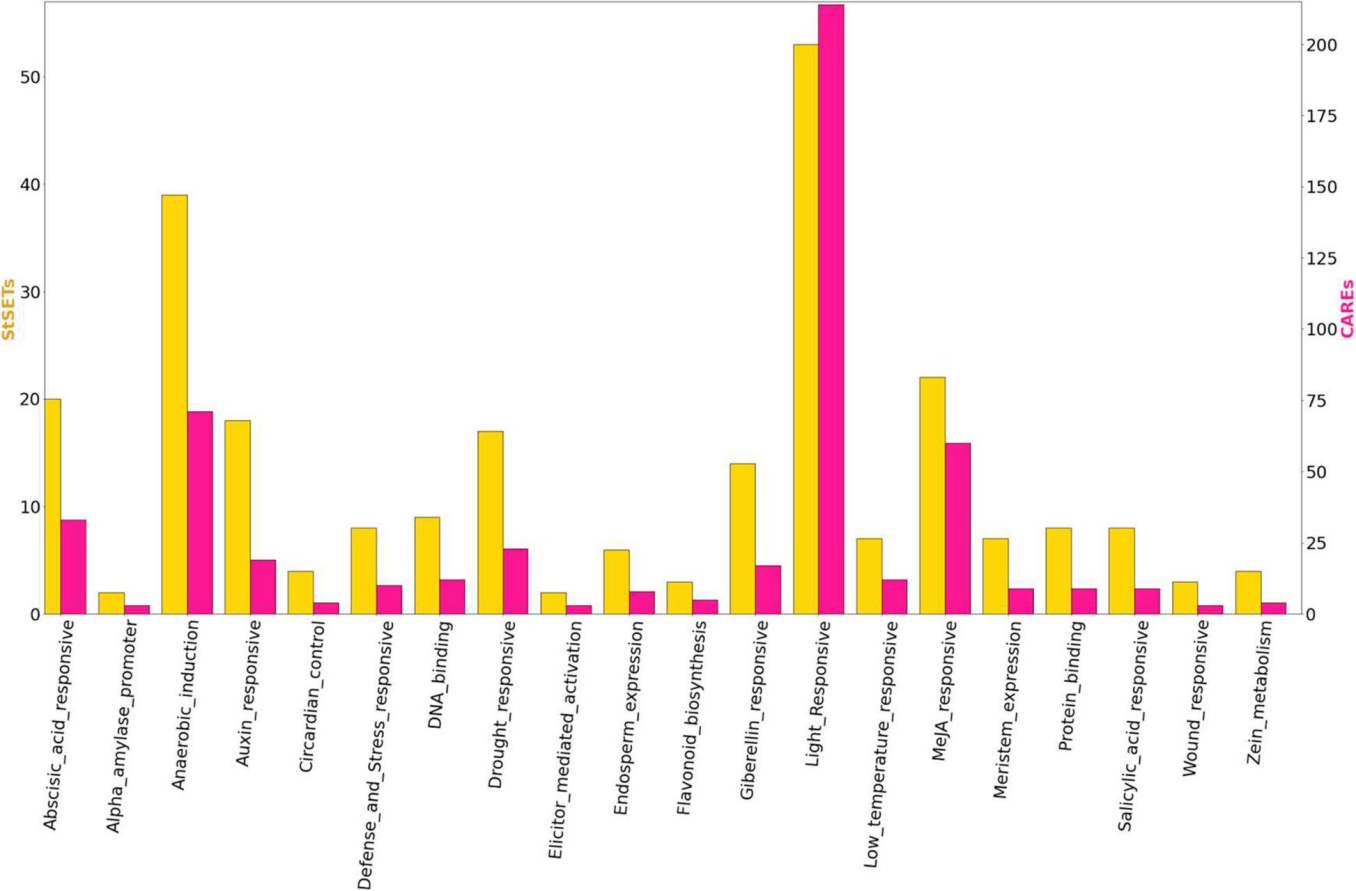
The *cis*-elements (CAREs) with a frequency >  = 3, detected within a 1 kb region upstream of the transcription start site. The yellow color bars indicate the number of genes in which respective *cis*-element is identified. The magenta color bars indicate the sum of respective *cis*-elements. We identified *cis*-elements within the promoter sequences using the PlantCARE database with a frequency cut-off of three for each *cis*-element [36]

We identified 41 unique *cis*-elements in the non-overlapping 1 Kb region upstream (potential promoter sequence) to the transcription start site of StSET genes (Table 3; Table S4). Among these, we identified several *cis*-elements described previously in the context of various environmental factors. For example, the promoter sequences of 53 StSET genes contained *cis*-elements described previously in the context of light-responsiveness. In addition, we found several drought-responsive, abscisic acid-, salicylic acid-, methyl jasmone acid- and auxin-responsive elements (Fig. 4). In addition, we identified 20 conserved motifs with a length range of 28—100 nucleotides within the potential promoter sequences of StSET genes (Table S5). Motifs 7 and 2 were conserved in 44 and 32 StSET genes, respectively, while motifs 1, 8, and 15 were conserved in two StSET genes (Table S5).

**Table 3:** List of *cis*-elements identified in promoter sequences of StSET genes. The sequence column indicates the *cis*-element identified in the promoter sequences. The count column indicates the number of *cis*-elements identified in promoter sequences across the StSET genes. The genes column indicates the number of StSET genes in which a specific *cis*-element is identified

Cis-element name	Sequence	Description	Count	Genes
ARE	AAACCA	Anaerobic induction	71	39
Box 4	ATTAAT	Light Responsive	70	35
ABRE	ACGTG	Abscisic acid responsive	33	20
CGTCA-motif	CGTCA	MeJA responsive	30	22
TGACG-motif	TGACG	MeJA responsive	30	22
GT1-motif	GGTTAA	Light Responsive	25	21
TCT-motif	TCTTAC	Light Responsive	24	19
MBS	CAACTG	Drought responsive	23	17
AuxRR-core	GGTCCAT	Auxin responsive	13	12
AT-rich element	ATAGAAATCAA	DNA binding	12	9
LTR	CCGAAA	Low-temperature responsive	12	7
ATCT-motif	AATCTAATCC	Light Responsive	11	10
TCCC-motif	TCTCCCT	Light Responsive	11	10
MRE	AACCTAA	Light Responsive	10	10
TC-rich repeats	GTTTTCTTAC	Defense and Stress responsive	10	8
CAT-box	GCCACT	Meristem expression	9	7
CCAAT-box	CAACGG	Protein binding	9	8
TCA-element	CCATCTTTTT	Salicylic acid responsive	9	8
AE-box	AGAAACAA	Light Responsive	8	7
GCN4_motif	TGAGTCA	Endosperm expression	8	6
P-box	CCTTTTG	Gibberellin responsive	8	8
chs-CMA1a	TTACTTAA	Light Responsive	8	7
G-box	CACGTG	Light Responsive	7	5
LAMP-element	CTTTATCA	Light Responsive	7	7
AT1-motif	AATTATTTTTTATT	Light Responsive	6	6
GA-motif	ATAGATAA	Light Responsive	6	6
TGA-element	AACGAC	Auxin responsive	6	6
MBSI	aaaAaaC(G/C)GTTA	Flavonoid biosynthesis	5	3
TATC-box	TATCCCA	Gibberellin responsive	5	4
Box II	TGGTAATAA	Light Responsive	4	4
GARE-motif	TCTGTTG	Gibberellin responsive	4	4
Gap-box	CAAATGAA(A/G)A	Light Responsive	4	4
I-box	GTATAAGGCC	Light Responsive	4	4
O2-site	GATGATGTGG	Zein metabolism	4	4
circadian	CAAAGATATC	Circadian control	4	4
A-box	CCGTCC	Alpha-amylase promoter	3	2
AT-rich sequence	TAAAATACT	Elicitor-mediated activation	3	2
GATA-motif	AAGATAAGATT	Light Responsive	3	3
Sp1	GGGCGG	Light Responsive	3	3
WUN-motif	AAATTTCCT	Wound responsive	3	3
chs-CMA2a	TCACTTGA	Light Responsive	3	3

### Tissue-specific expression of StSET genes

**Fig. 5:**
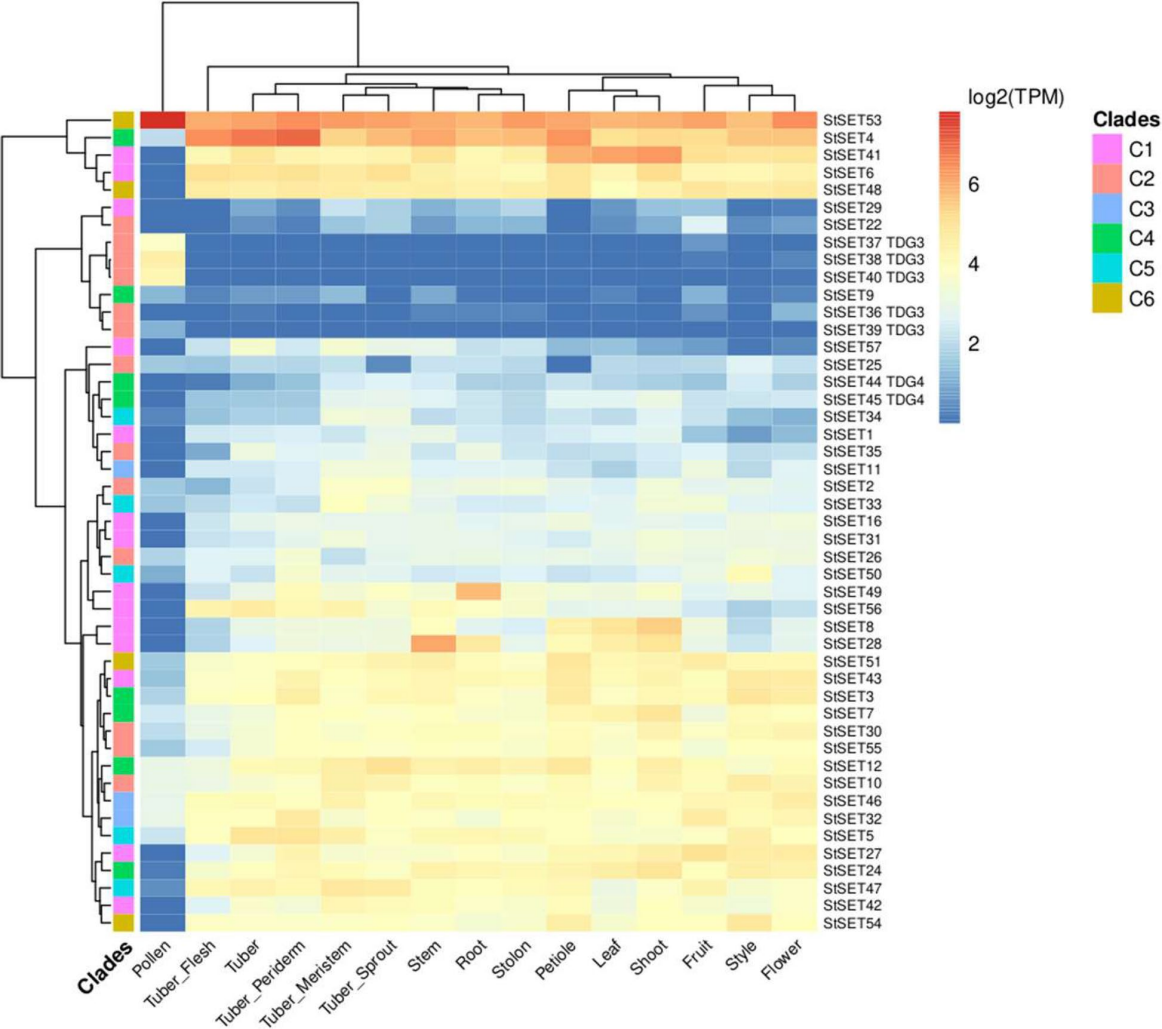
Global expression patterns of StSET genes in fifteen different tissues. Three genes, such as StSET37, StSET38, and StSET40, showed tissue-specific expression in pollen with an average Tau index of 0.9928. The expression values are log-transformed transcripts per million (TPM). The TPM values are retrieved from StCoExpNet [37]. Clades indicate the phylogenetic clades present in Fig. 2A. Tandemly duplicated StSET genes are labelled with corresponding cluster names (TDG3 & TDG4)

We investigated the expression patterns of all the identified StSET genes in 15 tissues, namely pollen, style, flower, fruit, leaf, petiole, stem, shoot, root, stolon, tuber, tuber meristem, tuber periderm, tuber flesh, and tuber sprout using the expression data retrieved from the StCoExpNet database [37]. A detectable expression, i.e., an average transcript per million (TPM) > 1 across samples of respective tissues, was observed in at least one tissue for 47 out of 57 StSET genes (Fig. 5). In addition, we found that about 84% of the StSET genes were assigned to 27 different co-expression clusters. We observed variation in expression between members of a phylogenetic clade and across phylogenetic clades (Figure S3). We found that seven of 13 tandemly duplicated StSET genes were expressed at least in one tissue. Moreover, three StSET genes belonging to TDG3, StSET37, StSET38, and StSET40, showed tissue-specific expression in pollen with an average Tau index of 0.9928 (Fig. 5; Table S6). In contrast, the remaining two genes, StSET36 and StSET39, revealed a low expression across tissues but showed slightly increased expression in flower and pollen, respectively. Two genes of TDG4, StSET44 and StSET45, were expressed in all tissues except pollen (Fig. 5; Table S6).

### Expression profiling of StSET genes in response to abiotic stress conditions

We investigated the relative expression of four StSET genes (StSET13, StSET30, StSET48 and StSET52) in three different potato genotypes: Karlena (drought-tolerant), Kolibri (drought-tolerant), and Laura (drought-sensitive and heat-tolerant), under drought and heat stress. We examined two-time points—9 days (T3) and 18 days (T6) in stress—plus four days after recovery (T7) for expression analysis.

**Fig. 6:**
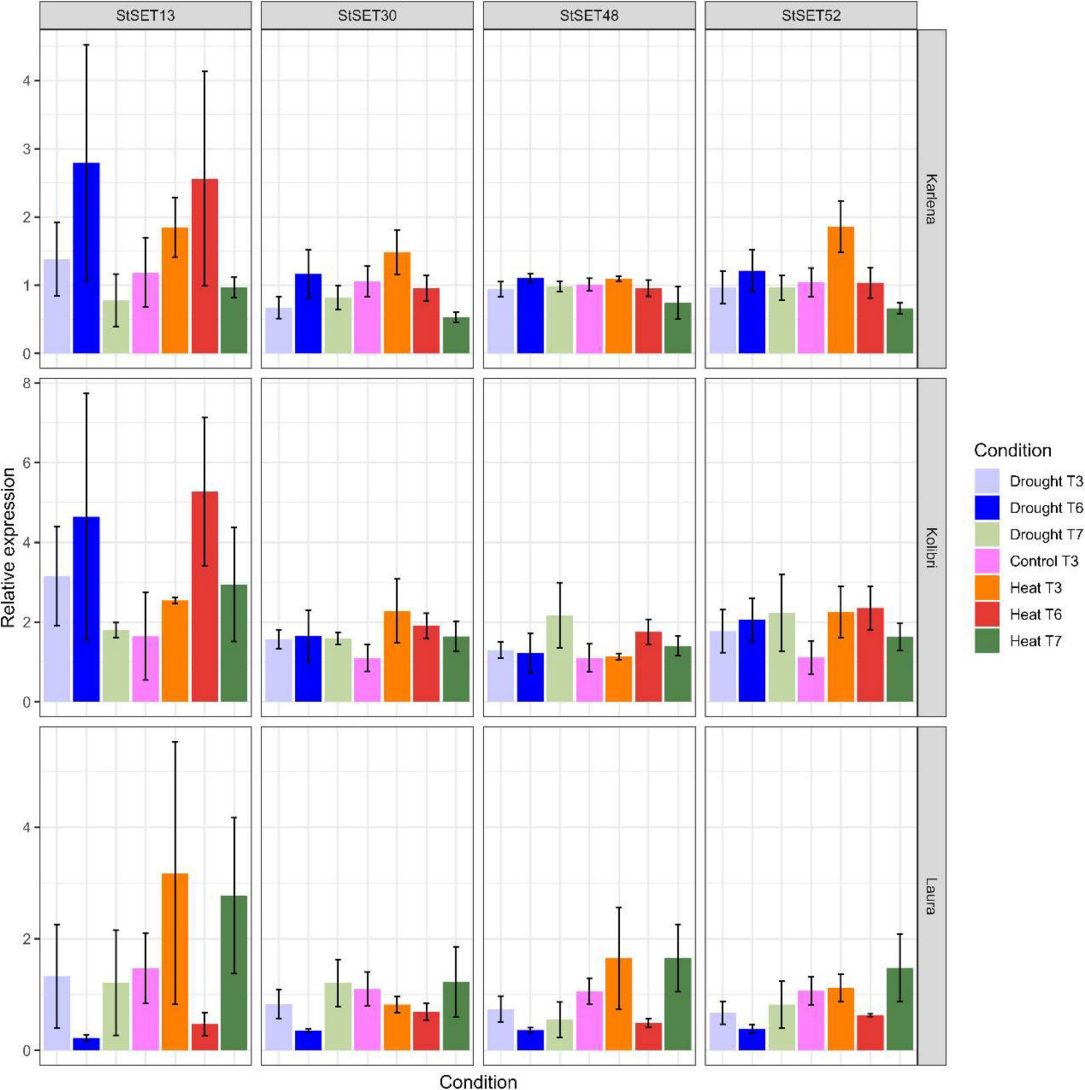
Expression profiling of StSET genes in response to abiotic stress treatments. Relative gene expression of four StSET genes analysed by RT-qPCR in response to drought and heat stress conditions in three potato clones. Karlena and Kolibri are drought-tolerant genotypes. Laura is drought-sensitive and heat-tolerant. The bar represents mean ± standard error (*n* = 3). T3 and T6 indicate that the RNA was sampled on the 9th and 18th day of respective stress conditions, while T7 indicates that the RNA was sampled on the fourth day after the recovery phase. The control T3 indicates that the RNA was sampled on the ninth day from the control plants

The RT-qPCR results showed a change in expression for all four genes under heat and drought stress in a genotype dependent manner. Compared to the control conditions the relative expression of all four genes increased 9 days after heat stress. Under prolonged exposure to heat stress (18 days), the relative expression of the investigated genes went down in Laura (heat tolerant) while the reverse was true for Karlena and Kolibri (heat sensitive) (Fig. 6). Compared to the control conditions, the relative expression of all StSET genes decreased under extreme drought stress (18 days after stress, 2.8% VMC). The relative expression of either gene increased (StSET13, StSET52) or remained at similar levels (StSET30 and StSET48) compared to control conditions in drought tolerant cultivars. The relative expression all four genes was comparable with those in control conditions after stress release in all cultivars (Fig. 6).

### Comparative analysis of SET domain-containing genes

**Fig. 7:**
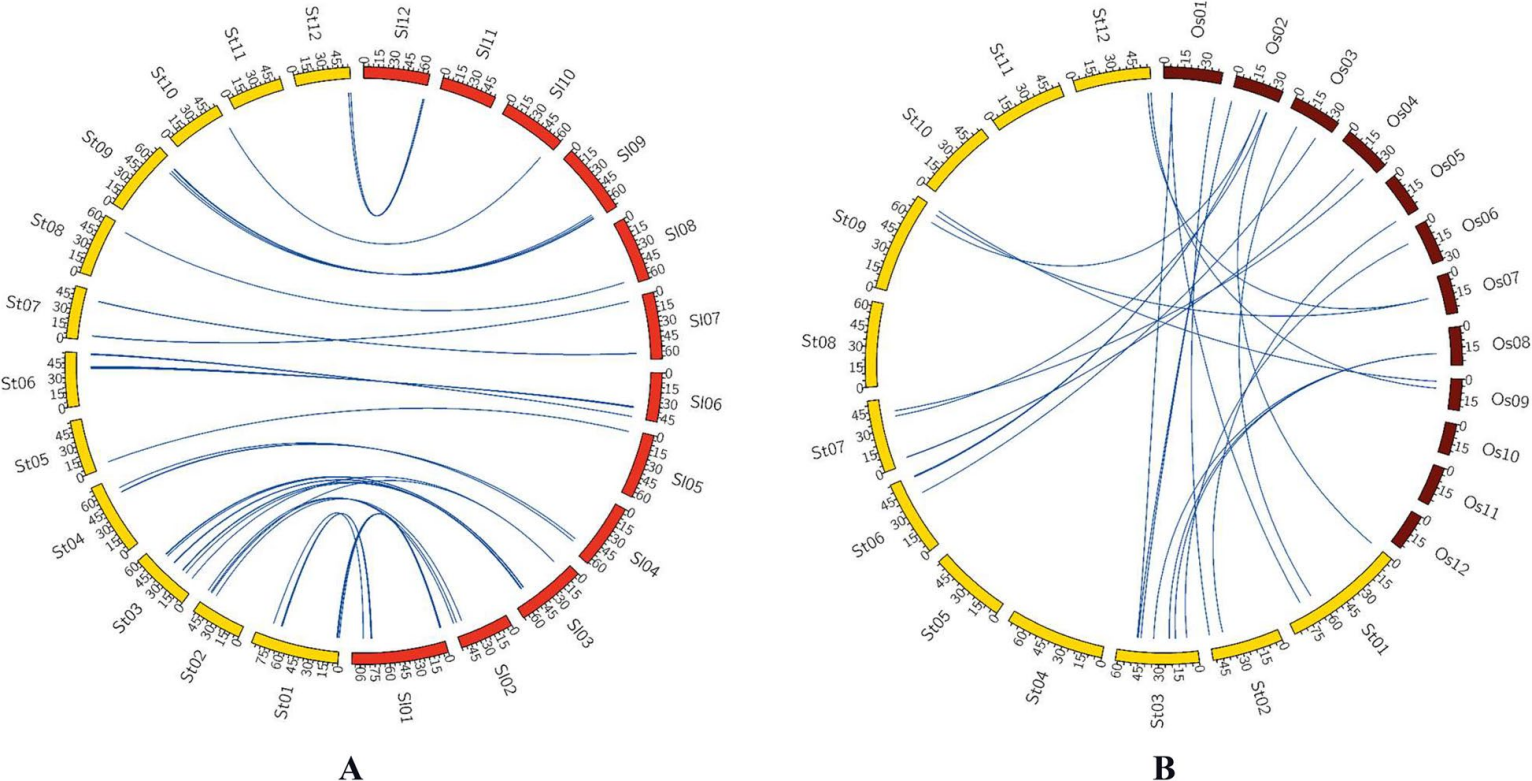
A comparative physical map of orthologous SET-domain containing genes among potato and other plant species visualised using CIRCOS v0.69–8 [38]. The comparative physical map between A) Potato and *Solanum lycopersicum*, and B) Potato and *Oryza sativa*

To derive orthologous relationships of StSET genes, a comparative mapping approach was followed wherein we compared the physically mapped SET domain-containing genes of potato with those of nine other species, namely *Arabidopsis thaliana*, *Camellia sinensis*, *Gossypium raimondii*, *Malus domestica*, *Oryza sativa*, *Populus trichocarpa*, *Setaria italica*, *Solanum lycopersicum*, and *Triticum aestivum*. In this study, we defined the orthologous SET domain-containing genes between potato and the species mentioned earlier based on the following criteria: If the SET domain-containing genes of the other nine species showed at least 50% of sequence identity and query coverage against StSET in BLASTP search, they were considered orthologs to StSETs. Based on these criteria, we observed a considerable variation in the number of orthologous SET domain-containing genes between potato and the species mentioned earlier (Table S7). For example, *Solanum lycopersicum* contained the highest number (about 79%) of orthologous SET domain-containing genes with potatoes. In contrast, *Oryza sativa* contained the lowest number (about 37%) of orthologous SET domain-containing genes with potatoes (Table S7; Fig. 7).

We observed the presence and absence of protein domains in SET domain-containing genes between potato and three other species (Table 1). For example, the protein domain, TDBD (PFAM ID: PF16135), was identified only in potato. In contrast, the protein domain, GYF_2 (PFAM ID: PF14237), was not detected in potato. Further, we observed the presence and absence of a unique combination of protein domains in SET domain-containing genes between potatoes and three other species (Table 2). For example, the protein domain combination, SET, Pre-SET, SAD_SRA, and Iso_dh, was only identified in potato, while the protein domain combination, SET, SAD_SRA, was absent in potato.

## Discussion

### SET domain-containing gene family in potato

SET domain-containing proteins that catalyse histone methylation on lysine residues are vital players for dynamically regulating the chromatin condensation [39], which in turn is essential to regulating genes in various developmental and physiological processes, such as floral organogenesis [40], root development [41], seed development [42], and plant responses to abiotic stress conditions [5, 22, 24]. However, information about the gene family that comprises the SET domain in potatoes was missing. Therefore, identifying members of this gene family will aid in comprehending the epigenetic mechanism that regulates gene expression in potato and, thus, potentially contribute to the phenotypic variation of agronomically important traits.

We identified 57 StSET genes in the potato genome and systematically characterised them (Fig. 1; Table S1). The number of StSET genes significantly exceeded the number of SET domain-containing genes identified in other plant species, including the potato's closest relative species used in this study, *Solanum lycopersicum* [28]. However, the number observed for potato was lower than in three species, including *Triticum aestivum* [29] (Table S7). Variation in the number of SET domain-containing genes among the species used in this study reflects the lineage-specific expansion of the gene family [43]. Further, we observed a significant variation in the number of orthologous SET domain-containing genes between potato and other species used in this study (Table S7), which is in accordance with the phylogenetic distance between potato and other species [44].

Our study identified six clades for SET domain-containing genes (Fig. 2A). This number is inconsistent with the number of clades identified in SET domain-containing genes of *Solanum lycopersicum* (Table S7), which belongs to the same genus as potato [28]. Although a phylogenetic clade is well defined, the criteria and datasets used to infer the phylogenetic clade vary among studies, which explain the observed variation in the number of clades among species. Interestingly, our phylogenetic analysis using the combined list of the StSET genes and SET domain-containing genes of three other species, including *Solanum lycopersicum* [28], identified six clades (Fig. 3). This result supports that the six phylogenetic clades for StSET genes are acceptable, following the number of clades identified for SET domain-containing genes of *Malus domestica* [24], *Populus trichocarpa* [26], and *Triticum aestivum* [29] (Table S7).

The analysis of *cis*-elements in the promoter regions allowed the prediction of potential mechanisms of StSET gene regulation. Our results showed that a diverse set of *cis*-elements were present in most StSET genes (Table 3), indicating that the StSET genes are involved in several diverse biological processes, including drought [45], anaerobic induction, auxin responsiveness, defense, stress responsiveness, wound, and low-temperature responsiveness (Fig. 4). Further, most of these *cis*-elements were reported to be present in the promoters of SET domain-containing genes of other plant species as well, including *Triticum aestivum* [29]*, Solanum lycopersicum* [28], *Oryza sativa* [25], and *Arabidopsis thaliana* [21], indicating the conservation of regulatory mechanism to control various biological processes mentioned above across species.

### Tandem duplication marginally contributes to the expansion of StSET genes

The duplication of genes has played a substantial role in eukaryotic evolution by contributing significantly to the genetic and morphological diversity and speciation [46]. Two whole-genome duplication events have occurred during potato genome evolution [47], and they generated tandemly duplicated genes (about 18% of genes) by sub-functionalisation and neo-functionalisation in the potato reference genome [48]. In this study, we found that about 23% of StSET genes were tandemly duplicated (Fig. 1; Table 4). As this rate is slightly higher than the genome-wide duplication rate, no particular expansion can be reported due to tandem duplication of the StSET genes. We observed similar expression patterns between StSETs of the same tandemly duplicated gene cluster while dissimilar expression patterns between members of different tandemly duplicated gene clusters was observed (Fig. 5). Further, we found that most tandemly duplicated StSET genes contained identical protein domains, showed an expression divergence across tissues (Fig. 5), and a neutral or purifying selection, i.e., Ka/Ks < 1 (Table 4). Altogether, these results indicate that retention of duplicated StSET genes occurred through sub-functionalisation, where the ancestral gene functions have become divided among the daughter copies, and both daughter copies must remain functional [48]. The proportion of tandemly duplicated StSET genes in potato is significantly higher than those identified in *Setaria italica* [27] and *Malus domestica* [24], which indicates the expansion of a gene family with tandemly duplicated genes in one species lineage tends to be coupled with losses in the other due to lineage-specific selection of tandemly duplicated genes [49].

**Table 4:** List of tandemly duplicated gene (TDG) clusters identified in StSET genes. Gene 1 and Gene 2 indicates a pair of tandemly duplicated genes. Ka and Ks indicate the number of non-synonymous substitutions per non-synonymous sites and synonymous substitutions per synonymous sites, respectively. Ka/Ks indicates the ratio of Ka and Ks. Time indicates the estimated time of divergence for tandem duplicated gene pairs calculated based on the Ka/Ks ratio

TDG cluster name	Gene 1	Gene 2	Ks	Ka	Ka/Ks
TDG1	StSET14	StSET15	0.4453	0.443	0.9948
TDG2	StSET18	StSET19	0.1278	0.0791	0.6186
TDG2	StSET18	StSET20	0.1522	0.0586	0.385
TDG2	StSET18	StSET21	0.153	0.0657	0.4295
TDG2	StSET19	StSET20	0.2577	0.1336	0.5183
TDG2	StSET19	StSET21	0.1944	0.1102	0.5669
TDG2	StSET20	StSET21	0.166	0.065	0.3916
TDG3	StSET36	StSET37	0.7288	0.4457	0.6116
TDG3	StSET36	StSET38	0.4436	0.341	0.7688
TDG3	StSET36	StSET39	0.5128	0.3205	0.625
TDG3	StSET36	StSET40	0.4702	0.3339	0.7101
TDG3	StSET37	StSET38	0.1138	0.0538	0.4723
TDG3	StSET37	StSET39	0.1146	0.099	0.8639
TDG3	StSET37	StSET40	0.1133	0.0892	0.7873
TDG3	StSET38	StSET39	0.4328	0.2283	0.5275
TDG3	StSET38	StSET40	0.1144	0.054	0.4722
TDG3	StSET39	StSET40	0.1453	0.105	0.7221
TDG4	StSET44	StSET45	0.0144	0.0128	0.8891

### Presence and absence of protein domains in StSET genes

Understanding protein domains is crucial for comprehending proteins' biological functions and evolutionary mechanisms, as they are the fundamental units that can function and evolve independently [50]. Thus, we performed a comparative analysis of protein domains identified in StSET genes against SET domain-containing genes of three species to identify the presence and absence variation of protein domains. The protein domain analysis highlighted the absence of three protein domains, such as AT hook (PFAM ID: PF02178), DUF4371 (PFAM ID: PF14291), and GYF_2 (PFAM ID: PF14237), in StSET genes (Fig. 2C; Table 1). In contrast, the StSET genes contain several protein domains absent in SET domain-containing genes of *Solanum lycopersicum* [28], although the species belongs to the same genus as potato (Table 1). These results indicate the evolution of novel biological functions of StSET genes by incorporating new protein domains with the existing ones. For example, the study identified a new protein domain, TDBD (Tify domain binding domain) (PFAM ID: PF16135), in the StSET45 gene absent in other plant species (Fig. 2C, Table 1). This domain binds with the Tify domain of JAZ1 proteins to play a role in stress-related and growth-related signalling cascades [51].

Recombination effects, such as duplication, insertion, deletion, and transposition, mainly determine the emergence of different domain combinations within proteins [52, 53]. The evolutionary selection of the newly created domain combinations is then influenced by the functional advantage it provides to the organism [54]. Thus, identifying novel protein domain combinations helps to better understand SET domain-containing proteins’ biological functions. In this study, we identified several protein domain combinations within SET domain-containing proteins across species (Fig. 2C; Table 2). For example, the SET domain-containing proteins of potato (StSET52) and *Oryza sativa* [25] comprised a unique combination of eight protein domains, such as SET, PWWP, FYRN, FYRC, PHD, PHD_2, zf-HC5HC2H_2, and zf-HC5HC2H. In contrast, this combination is absent from *Solanum lycopersicum* [28] and *Arabidopsis thaliana* [21]. Similarly, a unique combination of seven protein domains, such as SET, zf-HC5HC2H_2, zf-HC5HC2H, FYRN, FYRC, PWWP, and PHD_2, was identified in two SET domain-containing proteins of *Arabidopsis thaliana* [21], while being absent in other species, including in potato (Fig. 2C; Table 2). The SET domain-containing proteins with a unique combination of multiple protein domains might be involved in several biological processes in addition to catalysing the histone methylation on lysine residues in respective species.

### Pollen-specific expression of StSET genes

Due to the critical roles of SET domain-containing genes in various plant developmental processes, the expression of these genes in different tissues has been studied in many species, including *Solanum lycopersicum* [28], *Setaria italica* [27], and *Triticum aestivum* [29]. We observed the expression of about 82% of StSET genes in at least one tissue (Fig. 5). In addition, most of the genes showed a high expression in all tissues except pollen and indicated key roles of SET domain-containing genes in diverse tissues. Notably, three tandemly duplicated genes, namely StSET37, StSET38, and StSET40, exhibited a pollen-specific gene expression with an average Tau index of 0.9928 (Fig. 5; Table S6). The tissue-specific expression of StSET genes in pollens might indicate that these genes are involved in pollen development. For example, SDG4, a SET domain-containing gene, regulates the pollen tube growth by methylation of histone H3 lysines 4 and 36 in mature pollens of *Arabidopsis thaliana* [55]. In addition, our study highlights the expression divergence across tissues between members of a phylogenetic clade and across phylogenetic clades (Figure S3).

### Expression profiling of StSET genes in response to abiotic stress

Recent studies suggest that SET domain-containing genes are involved in plant stress responses [16, 22, 24, 27]. Thus, we assessed the expression patterns of four candidate StSET genes, StSET13, StSET30, StSET48, and StSET52, under heat and drought stresses using RT-qPCR in three potato clones. The RT-qPCR results showed that the four candidate StSET genes showed extensive variation in drought and heat-stress-induced gene expression changes across three potato genotypes: Karlena and Kolibri (drought-tolerant), and Laura (drought-sensitive and heat-tolerant).

StSET13 exhibited a consistent pattern of increased expression from early (T3) to late-stage (T6) drought stress in the drought-tolerant genotypes Karlena and Kolibri. This trend, however, was not observed in the drought-sensitive genotype Laura where the relative expression was even down-regulated compared to control conditions under extreme drought. This observation indicated a potential difference in their genotype-specific stress response mechanisms under drought (Fig. 6). Similarly, drought-induced SET domain-containing genes have been reported in many plant species. For example, 21 SET domain-containing genes exhibited a differential expression in response to drought stress in a drought-tolerant *Setaria italica* genotype [27].

Under heat stress, all four genes showed high expression during the early stage of heat stress (T3) in the heat-tolerant genotype Laura. In comparison, all four genes showed high expression during either early or late-stage heat stress in drought-tolerant genotypes Karlena and Kolibri, respectively (Fig. 6), suggesting a potential difference in their genotype-specific stress response mechanisms under varying durations of heat stress. Similarly, SET domain-containing genes of *Gossypium raimondii* showed differential expression in response to heat stress to affect the methylation status of stress-responsive genes to further regulate in response to heat stress [23].

Furthermore, most of these genes showed a decline in expression after recovering from the stress (Fig. 6). The high expression of StSET genes under stress and a decline during recovery from stress might be caused by the histone modifications regulating various stress-responsive genes to withstand the abiotic stress, followed by reverting histone modifications to their normal levels once the stressor is no longer present [17, 56, 57]. Therefore, StSET genes may be crucial in conferring drought and heat stress tolerance in potatoes. It is important to validate the observed expression patterns through independent experiments and analyses, such as using additional genotypes, different abiotic stress conditions or complementary techniques like RNA-Seq.

## Conclusion

In conclusion, this study provides valuable insights into the SET gene family in *Solanum tuberosum*. We identified a total of 57 StSET genes in the potato genome, with a majority of StSET genes distributed among 11 chromosomes. Phylogenetic analysis classified the structurally diverse StSET genes into six groups. Gene duplication analysis indicated that tandem duplication played only a marginal role in the expansion of StSET genes. We examined the distinct protein domain combinations of the SET domain and other protein domains and compared them between potato and other plant species. We performed in silico tissue-specific expression analysis of StSET genes among 15 potato tissues to unravel their biological activity in different organs. RT-qPCR assessed the expression profiles for StSET genes under abiotic stress conditions to infer their genetic role in stress tolerance. Overall, this study presents a comprehensive analysis of the SET gene family in potato and will contribute to further characterization and elucidation of the epigenetic regulatory mechanisms of the SET gene family in different potato genotypes and related plant species.

## Materials & methods

### Identification of StSET genes in *Solanum tuberosum*

The protein sequences of SET domain-containing genes reported in *Arabidopsis thaliana* [21], *Camellia sinensis* [22], *Gossypium raimondii* [23]*, Malus domestica* [24], *Oryza sativa* [25]*, Populus trichocarpa* [26]*, Setaria italica* [27]*, Solanum lycopersicum* [28] and *Triticum aestivum* [29], were retrieved and used as input sequences to identify StSET genes in potato using sequence- and profile-based approaches. Here, we used the genomic sequence and annotation data of the diploid clone derived from the potato cultivar Agria (dAg) [48, 58] as a reference genome to identify the SET domain-containing genes in potato. In the sequence-based approach, the above-retrieved protein sequences were searched against the protein sequences of dAg using BLASTP [59] with an e-value cut-off of 1e^−10^. In the profile-based approach, we computed a multiple-sequence alignment (MSA) using the above-retrieved protein sequences using ClustalW with the default parameters [60]. We created a Hidden-Markov Model (HMM) profile by feeding the above-computed MSA to hmmbuild with the default parameters [61], and we searched for StSET genes in the protein sequences of dAg using hmmsearch with the default parameters [61] with an e-value cut-off of 1e^−10^ using the above-computed HMM profile as a query. Finally, we combined the list of putative StSET genes obtained from both approaches. We fed the corresponding protein sequences of the unique putative StSET genes to InterProScan [62] and Pfam [63] to confirm the presence of the SET domain (InterProScan ID: IPR001214; PFAM ID: PF00856). In addition, we have performed a BLASTP search [59] using the confirmed StSET protein sequences as a query against the proteome of dAg to identify additional SET domain-containing proteins in the reference genome except those obtained by the above search. BLASTP [59] hits with e-value cut-off of 1e^−10^ and >  = 50% of sequence similarity and query coverage were fed to InterProScan [62] and Pfam [63] to confirm the presence of the SET domain (InterProScan ID: IPR001214; PFAM ID: PF00856). Overrepresented gene ontology terms were identified for identified StSET genes using WEGO 2.0 [64].

### Physical mapping, gene structure, and domain organisation of StSET genes

We extracted the chromosomal location of individual StSET genes from the annotation (gff) of the potato reference genome [48]. We visualised the physical mapping of StSET genes using MapChart v2.32 [33]. We extracted the coordinates of the exon, intron, and UTR regions of individual StSET genes from the annotation of dAg, and we visualised the gene structure as well as protein domain organisation using TBTools v1.098696 [34].

### Physicochemical properties, sub-cellular location, and trans-membrane regions of StSET genes

We computed the physicochemical properties of StSET genes by submitting the protein sequences of StSET genes to ProtParam (https://web.expasy.org/protparam). We predicted the sub-cellular localisation of StSET genes by submitting the protein sequences of StSET genes to the SignalP v6 [65] and TargetP v2 [66] web servers. We predicted the transmembrane regions of StSET genes by submitting the protein sequences of StSET genes to the TMHMM server [67].

### Identification of conserved motifs and cis-elements in promoters of StSET genes

We retrieved the non-overlapping 1 Kb length sequence upstream of the transcription start site (TSS) for each StSET gene from the genome sequences of dAg [58] and considered it the putative promoter sequence. Using the MEME Suite web server [68], we identified the top 20 conserved motifs in the promoter sequences of StSET genes. The parameters used were motif width: 5 to 100 bases; site distribution: any number of repetitions. We identified *cis*-elements within the promoter sequences using the PlantCARE database with a frequency cut-off of three for each *cis*-element [36].

### Identification of duplicated StSET genes

We identified duplicated StSET genes by performing an all versus all BLASTP search between protein sequences of all StSET genes, followed by feeding the BLASTP results to MCScanX with the default parameters [69]. We aligned the protein sequences of each pair of duplicated StSET genes using MAFFT v7.453 with the default parameters [70], and we calculated the non-synonymous (Ka) and synonymous (Ks) substitutions and their ratios (Ka/Ks) using PAL2NAL with the default parameters [71]. Finally, we highlighted the duplicated StSET genes on the physical mapping of StSET genes created earlier.

### Phylogenetic analysis of StSET genes

We computed an MSA for StSET genes using respective protein sequences by feeding to the MAFFT program v7.453 [70] with default parameters. We computed a mid-rooted phylogenetic tree for StSET genes by feeding the above-computed MSA to RAxML v8.2.12 [72] with the PROTGAMMAAUTO and JTT models and 100 iterations. Similarly, we computed a phylogenetic tree by feeding the protein sequences of StSET genes and SDGs reported in *Arabidopsis thaliana* [21], *Oryza sativa* [25], and *Solanum lycopersicum* [28]. We visualised the computed phylogenetic trees using TBTools [34] and iTol [35] and classified the SET genes based on their phylogenetic clade membership.

### In silico tissue-specific expression profiling of StSET genes

We performed gene expression analysis of the identified StSET genes across fifteen tissues using the expression data available in the StCoExpNet database [37]. Further, we assessed the tissue specificity of the identified StSET genes using the tissue-specificity index (Tau) using the same database.

### Plant materials and abiotic stress treatments

Five tetraploid potato cultivars, namely Agria, Jelly, Karlena (drought-tolerant) [73], Kolibri (drought-tolerant) [73] and Laura (drought-sensitive and heat-tolerant) [74], were grown in plant growth chambers (Fitotron SGC 120 Humidity, Weiss Technik GmbH, Germany) in 1.5 L pots using a peat-based potting mixture ED73 classic (Einheitserde, Germany). We set the light intensity to ~ 400 μmol m^−2^ s^−1^, the day/night temperature to 22° C/20° C, and the relative humidity to 70%. Shortly before the stress experiment started, we brought the pots with the same volumetric moisture content (VMC) of ~ 50%. During the stress phase, we controlled the VMC daily using a moisture meter sensor (SM150T, DeltaT devices, United Kingdom). We determined the linear regression between VMC and gravimetric moisture content (%) for watering the pots to 50% VMC (Figure S1). The results of two potato cultivars, Agria and Jelly, were excluded from the experiment due to a technical problem in the plant growth chamber after a few weeks of plant growth. We subjected five-week-old potato plants to drought and heat stress, as described below.

Drought stress was applied by controlled dehydration, ensuring a uniform decrease in VMC across all the pots under water stress. The depletion rate in VMC stabilized seven days after the start of dehydration. The mean VMC of pots under drought stress on T3 and T6 was 7.6% and 2.8%, respectively. After 18 days, the recovery phase started, and we rewatered the drought-stressed plants to realize 50% VMC. We exposed the plants for two weeks to heat stress (day/night temperature of 35° C/28° C). The plants were daily watered up to keep 50% VMC during heat stress. After 18 days, the recovery phase started, and the heat-stressed plants were grown under the same temperatures as the control conditions. All experiments were performed in three biological replicates for control, drought, and heat stresses.

We collected leaf samples nine (T3) and eighteen days (T6) after the start of the stress treatment, and the final sampling was performed four days after the recovery phase (T7). The samples were snap-frozen in liquid nitrogen and stored at -80 °C before further processing.

### RNA extraction and quantitative real time quantitative PCR (RT-qPCR) of StSET genes

Total RNA was isolated from the frozen leaf samples using the RNeasy Plant Mini Kit (Qiagen, Germany), following the manufacturer’s instructions, including RNase-free DNase I treatment. The RNA integrity and purity were evaluated using a NanoDrop spectrophotometer (Thermo Fischer Scientific, USA). Next, we synthesised the first strand of cDNA from total RNA (1500 ng) using the LunaScript™ RT SuperMix (New England Biolabs, USA). The real-time quantitative PCR (RT-qPCR) reaction was prepared using the Luna Universal RT-qPCR Master Mix Kit (New England Biolabs, USA) and the reaction was performed on the QuantStudio™ 5 Real-Time PCR system (Thermo Fischer Scientific, USA) in two technical replicates for each biological replicate. The reactions were carried out using the following parameters: 95 °C for 3 min, 40 cycles of 15 s at 95 °C, and 1 min at 60 °C, followed by 15 s at 95 °C for melting curve analysis. We designed gene-specific primers using the PrimerQuest tool (https://eu.idtdna.com/pages/tools/primerquest) for four StSET genes, StSET13, StSET30, StSET48 and StSET52. We selected these four genes based on the criteria that each gene should belong to a unique phylogenetic clade and contain a unique combination of protein domains. A constitutive Importin subunit alpha (StAlpha) gene-based primer was used as endogenous control [75]. The efficiency of primer pairs was between 84.47 to 93.91% (Table S8). We used Control T3 as an endogenous control and computed the relative expression for all four genes during stress (T3 and T6 time points) and after the recovery phase (T7). The relative gene expression level of four StSET genes was computed following Pfaffl (2001) [76].

### Comparative analysis of StSET genes

We identified the orthologous SET domain-containing genes between potato and nine other species, such as *Arabidopsis thaliana* [21]*, Camellia sinensis* [22], *Gossypium raimondii* [23]*, Malus domestica* [24]*, Oryza sativa* [25]*, Populus trichocarpa* [26]*, Setaria italica* [27]*, Solanum lycopersicum* [28] and *Triticum aestivum* [29]*,* using BLASTP [59]. If the SET domain-containing genes of the other species mentioned above show at least 50% of sequence identity and query coverage against SET domain-containing genes of potato in BLASTP search, they are considered orthologs to potato’s SET domain-containing genes. We compared the physical mapping of SET domain-containing genes between potato and two selected species: *Oryza sativa* [25]*,* and *Solanum lycopersicum* [28]. We visualised the syntenic relationship of SET genes using Circos v0.69–8 [38]. Further, we compared the StSET genes against SET domain-containing genes of the above mentioned three selected species regarding the presence and absence of protein domains and protein domain combinations.

### Supplementary Information

Supplementary Material 1.

Supplementary Material 2.

## Data Availability

All data generated or analysed during this study are included in this published article and can be found in Supplementary Tables S1 – S7.

## References

[1] Kornberg RD (1974). Chromatin structure: a repeating unit of histones and DNA. Science (New York, N.Y.).

[2] Deal RB, Henikoff S (2011). Histone variants and modifications in plant gene regulation. Curr Opin Plant Biol.

[3] Pfluger J, Wagner D (2007). Histone modifications and dynamic regulation of genome accessibility in plants. Curr Opin Plant Biol.

[4] Asensi-Fabado MA, Amtmann A, Perrella G (2017). Plant responses to abiotic stress: the chromatin context of transcriptional regulation. Biochim Biophys Acta Gene Regul Mech.

[5] Kim JM, Sasaki T, Ueda M, Sako K, Seki M (2015). Chromatin changes in response to drought, salinity, heat, and cold stresses in plants. Front Plant Sci.

[6] Xiao J, Lee US, Wagner D (2016). Tug of war: adding and removing histone lysine methylation in Arabidopsis. Curr Opin Plant Biol.

[7] Park J, Lim CJ, Shen M, Park HJ, Cha JY, Iniesto E, Rubio V, Mengiste T, Zhu JK, Bressan RA, Lee SY, Lee BH, Jin JB, Pardo JM, Kim WY, Yun DJ (2018). Epigenetic switch from repressive to permissive chromatin in response to cold stress. Proc Natl Acad Sci USA.

[8] Liu X, Zhou C, Zhao Y, Zhou S, Wang W, Zhou DX (2014). The rice enhancer of zeste [E(z)] genes SDG711 and SDG718 are respectively involved in long day and short day signaling to mediate the accurate photoperiod control of flowering time. Front Plant Sci.

[9] Ng DW, Wang T, Chandrasekharan MB, Aramayo R, Kertbundit S, Hall TC (2007). Plant SET domain-containing proteins: structure, function and regulation. Biochem Biophys Acta.

[10] Dillon SC, Zhang X, Trievel RC, Cheng X (2005). The SET-domain protein superfamily: protein lysine methyltransferases. Genome Biol.

[11] Liu C, Lu F, Cui X, Cao X (2010). Histone methylation in higher plants. Annu Rev Plant Biol.

[12] Casas-Mollano JA, Zacarias E, Almeida J, Tollefsbol TO (2023). Evolution of epigenetic mechanisms in plants: insights from H3K4 and H3K27 methyltransferases. Handbook of Epigenetics.

[13] Marmorstein R (2003). Structure of SET domain proteins: a new twist on histone methylation. Trends Biochem Sci.

[14] Wei G, Liu K, Shen T, Shi J, Liu B, Han M, Peng M, Fu H, Song Y, Zhu J, Dong A, Ni T (2018). Position-specific intron retention is mediated by the histone methyltransferase SDG725. BMC Biol.

[15] Ding Y, Wang X, Su L, Zhai J, Cao S, Zhang D, Liu C, Bi Y, Qian Q, Cheng Z, Chu C, Cao X (2007). SDG714, a histone H3K9 methyltransferase, is involved in Tos17 DNA methylation and transposition in rice. Plant Cell.

[16] Liu Y, Zhang A, Yin H, Meng Q, Yu X, Huang S, Wang J, Ahmad R, Liu B, Xu ZY (2018). Trithorax-group proteins ARABIDOPSIS TRITHORAX4 (ATX4) and ATX5 function in abscisic acid and dehydration stress responses. New Phytol.

[17] Ding Y, Avramova Z, Fromm M (2011). The Arabidopsis trithorax-like factor ATX1 functions in dehydration stress responses via ABA-dependent and ABA-independent pathways. Plant J.

[18] Sun C, Fang J, Zhao T, Xu B, Zhang F, Liu L, Tang J, Zhang G, Deng X, Chen F, Qian Q, Cao X, Chu C (2012). The histone methyltransferase SDG724 mediates H3K36me2/3 deposition at MADS50 and RFT1 and promotes flowering in rice. Plant Cell.

[19] Dong G, Ma DP, Li J (2008). The histone methyltransferase SDG8 regulates shoot branching in Arabidopsis. Biochem Biophys Res Commun.

[20] Cazzonelli CI, Cuttriss AJ, Cossetto SB, Pye W, Crisp P, Whelan J, Finnegan EJ, Turnbull C, Pogson BJ (2009). Regulation of carotenoid composition and shoot branching in Arabidopsis by a chromatin modifying histone methyltransferase, SDG8. Plant Cell.

[21] Zhang LS, Ma CR, Ji Q, Wang YF (2009). Genome-wide identification, classification and expression analyses of SET domain gene family in Arabidopsis and rice. Yi Chuan.

[22] Chen Q, Hu S, Guo F, Zhao H, Wang M, Ni D, Wang Y, Wang P. Characterisation of the SET DOMAIN GROUP gene family members in Camellia sinensis and functional analysis of the SDG43 gene in abiotic stresses. Environm Exp Bot. 2021;182. 10.1016/j.envexpbot.2020.104306.

[23] Huang Y, Mo Y, Chen P, Yuan X, Meng F, Zhu S, Liu Z (2016). Identification of SET domain-containing proteins in Gossypium raimondii and their response to high temperature stress. Sci Rep.

[24] Li W, Yan J, Wang S, Wang Q, Wang C, Li Z, Zhang D, Ma F, Guan Q, Xu J (2021). Genome-wide analysis of SET-domain group histone methyltransferases in apple reveals their role in development and stress responses. BMC Genomics.

[25] Lu Z, Huang X, Ouyang Y, Yao J (2013). Genome-Wide Identification, Phylogenetic and Co-Expression Analysis of *OsSET* Gene Family in Rice. PLoS ONE.

[26] Lei L, Zhou SL, Ma H, Zhang LS (2012). Expansion and diversification of the SET domain gene family following whole-genome duplications in Populus trichocarpa. BMC Evol Biol.

[27] Yadav CB, Muthamilarasan M, Dangi A, Shweta S, Prasad M (2016). Comprehensive analysis of SET domain gene family in foxtail millet identifies the putative role of SiSET14 in abiotic stress tolerance. Sci Rep.

[28] AieseCigliano R, Sanseverino W, Cremona G, Ercolano MR, Conicella C, Consiglio FM (2013). Genome-wide analysis of histone modifiers in tomato: Gaining an insight into their developmental roles. BMC Genomics.

[29] Batra R, Gautam T, Pal S, Chaturvedi D, Rakhi, Jan I, Balyan HS, Gupta PK (2020). Identification and characterisation of SET domain family genes in bread wheat (Triticum aestivum L.). Sci Rep.

[30] Bao Z, Li C, Li G, Wang P, Peng Z, Cheng L, Li H, Zhang Z, Li Y, Huang W, Ye M, Dong D, Cheng Z, VanderZaag P, Jacobsen E, Bachem CWB, Dong S, Zhang C, Huang S, Zhou Q (2022). Genome architecture and tetrasomicinheritance of autotetraploid potato. Mol Plant.

[31] FAO. Statistical data. Rome. 2021.

[32] Demirel U, Çalişkan ME, Bakhsh A, Jabran K (2023). Environmental requirements of potato and abiotic stress factors. Potato Production Worldwide.

[33] Voorrips RE (2002). MapChart: software for the graphical presentation of linkage maps and QTLs. J Hered.

[34] Chen C, Chen H, Zhang Y, Thomas HR, Frank MH, He Y, Xia R (2020). TBtools: an integrative toolkit developed for interactive analyses of big biological data. Mol Plant.

[35] Letunic I, Bork P (2021). Interactive Tree Of Life (iTOL) v5: an online tool for phylogenetic tree display and annotation. Nucleic Acids Res.

[36] Lescot M, Déhais P, Thijs G, Marchal K, Moreau Y, Van de Peer Y, Rouzé P, Rombauts S (2002). PlantCARE, a database of plant cis-acting regulatory elements and a portal to tools for in silico analysis of promoter sequences. Nucleic Acids Res.

[37] Bonthala VS, Stich B (2024). StCoExpNet: a global co-expression network analysis facilitates identifying genes underlying agronomic traits in potatoes. Plant Cell Rep.

[38] Krzywinski M, Schein J, Birol I, Connors J, Gascoyne R, Horsman D, Jones SJ, Marra MA (2009). Circos: an information aesthetic for comparative genomics. Genome Res.

[39] Zhou H, Liu Y, Liang Y, Zhou D, Li S, Lin S, Dong H, Huang L (2020). The function of histone lysine methylation related SET domain group proteins in plants. Protein Sci.

[40] Chen LQ, Luo JH, Cui ZH, Xue M, Wang L, Zhang XY, Pawlowski WP, He Y (2017). ATX3, ATX4, and ATX5 encode putative H3K4 methyltransferases and are critical for plant development. Plant Physiol.

[41] Gu X, Xu T, He Y (2014). A histone H3 lysine-27 methyltransferase complex represses lateral root formation in Arabidopsis thaliana. Mol Plant.

[42] Pontvianne F, Blevins T, Pikaard CS (2010). Arabidopsis histone lysine methyltransferases. Adv Bot Res.

[43] Lespinet O, Wolf YI, Koonin EV, Aravind L (2002). The role of lineage-specific gene family expansion in the evolution of eukaryotes. Genome Res.

[44] Tulpan D, Leger S. The plant orthology browser: an orthology and gene-order visualizer for plant comparative genomics. Plant Genome. 2017;10(1). 10.3835/plantgenome2016.08.0078. 10.3835/plantgenome2016.08.0078 28464063

[45] Chen Q, Guo L, Yuan Y, Hu S, Guo F, Zhao H, Yun Z, Wang Y, Wang M, Ni D, Zhao L, Wang P (2021). Ectopic overexpression of histone H3K4 methyltransferase CsSDG36 from tea plant decreases hyperosmotic stress tolerance in *Arabidopsis thaliana*. Int J Mol Sci.

[46] Ohno S (1970). Evolution by gene duplication.

[47] Potato Genome Sequencing Consortium, Xu X, Pan S, Cheng S, Zhang B, Mu D, Ni P, Zhang G, Yang S, Li R, Wang J, Orjeda G, Guzman F, Torres M, Lozano R, Ponce O, Martinez D, De la Cruz G, Chakrabarti SK, Patil VU, …, Visser RG. Genome sequence and analysis of the tuber crop potato. Nature. 2011;475(7355):189–195. 10.1038/nature10158. 10.1038/nature10158 21743474

[48] Bonthala VS, Stich B (2022). Genetic divergence of lineage-specific tandemly duplicated gene clusters in four diploid potato genotypes. Front Plant Sci.

[49] Hanada K, Zou C, Lehti-Shiu MD, Shinozaki K, Shiu SH (2008). Importance of lineage-specific expansion of plant tandem duplicates in the adaptive response to environmental stimuli. Plant Physiol.

[50] Wang Y, Zhang H, Zhong H, Xue Z (2021). Protein domain identification methods and online resources. Comput Struct Biotechnol J.

[51] Pauwels L, Barbero GF, Geerinck J, Tilleman S, Grunewald W, Pérez AC, Chico JM, Bossche RV, Sewell J, Gil E, García-Casado G, Witters E, Inzé D, Long JA, De Jaeger G, Solano R, Goossens A (2010). NINJA connects the co-repressor TOPLESS to jasmonate signalling. Nature.

[52] Weiner J, Beaussart F, Bornberg-Bauer E (2006). Domain deletions and substitutions in the modular protein evolution. FEBS J.

[53] Xia Y, Levitt M (2002). Roles of mutation and recombination in the evolution of protein thermodynamics. Proc Natl Acad Sci USA.

[54] Chothia C, Gough J (2009). Genomic and structural aspects of protein evolution. Biochem J.

[55] Cartagena JA, Matsunaga S, Seki M, Kurihara D, Yokoyama M, Shinozaki K, Fujimoto S, Azumi Y, Uchiyama S, Fukui K (2008). The Arabidopsis SDG4 contributes to the regulation of pollen tube growth by methylation of histone H3 lysines 4 and 36 in mature pollen. Dev Biol.

[56] Kim JM, To TK, Ishida J, Matsui A, Kimura H, Seki M (2012). Transition of chromatin status during the process of recovery from drought stress in Arabidopsis thaliana. Plant Cell Physiol.

[57] Zong W, Zhong X, You J, Xiong L (2013). Genome-wide profiling of histone H3K4-tri-methylation and gene expression in rice under drought stress. Plant Mol Biol.

[58] Freire R, Weisweiler M, Guerreiro R, Baig N, Hüttel B, Obeng-Hinneh E, Renner J, Hartje S, Muders K, Truberg B, Rosen A, Prigge V, Bruckmüller J, Lübeck J, Stich B (2021). Chromosome-scale reference genome assembly of a diploid potato clone derived from an elite variety. G3 (Bethesda, Md.).

[59] Altschul SF, Gish W, Miller W, Myers EW, Lipman DJ (1990). Basic local alignment search tool. J Mol Biol.

[60] Thompson JD, Higgins DG, Gibson TJ (1994). CLUSTAL W: improving the sensitivity of progressive multiple sequence alignment through sequence weighting, position-specific gap penalties and weight matrix choice. Nucleic Acids Res.

[61] Eddy SR (2011). Accelerated profile HMM searches. PLoS Comput Biol.

[62] Jones P, Binns D, Chang HY, Fraser M, Li W, McAnulla C, McWilliam H, Maslen J, Mitchell A, Nuka G, Pesseat S, Quinn AF, Sangrador-Vegas A, Scheremetjew M, Yong SY, Lopez R, Hunter S (2014). InterProScan 5: genome-scale protein function classification. Bioinformatics (Oxford, England).

[63] Mistry J, Chuguransky S, Williams L, Qureshi M, Salazar GA, Sonnhammer ELL, Tosatto SCE, Paladin L, Raj S, Richardson LJ, Finn RD, Bateman A (2021). Pfam: the protein families database in 2021. Nucleic Acids Res.

[64] Ye J, Zhang Y, Cui H, Liu J, Wu Y, Cheng Y, Xu H, Huang X, Li S, Zhou A, Zhang X, Bolund L, Chen Q, Wang J, Yang H, Fang L, Shi C (2018). WEGO 2.0: a web tool for analyzing and plotting GO annotations, 2018 update. Nucleic Acids Res.

[65] Teufel F, AlmagroArmenteros JJ, Johansen AR, Gíslason MH, Pihl SI, Tsirigos KD, Winther O, Brunak S, von Heijne G, Nielsen H (2022). SignalP 6.0 predicts all five types of signal peptides using protein language models. Nature Biotechnol.

[66] AlmagroArmenteros JJ, Salvatore M, Emanuelsson O, Winther O, von Heijne G, Elofsson A, Nielsen H (2019). Detecting sequence signals in targeting peptides using deep learning. Life Sci Alliance.

[67] Krogh A, Larsson B, von Heijne G, Sonnhammer EL (2001). Predicting transmembrane protein topology with a hidden Markov model: application to complete genomes. J Mol Biol.

[68] Bailey TL, Johnson J, Grant CE, Noble WS (2015). The MEME Suite. Nucleic Acids Res.

[69] Wang Y, Tang H, Debarry JD, Tan X, Li J, Wang X, Lee T, Jin H, Marler B, Guo H, Kissinger JC, Paterson AH (2012). MCScanX: a toolkit for detection and evolutionary analysis of gene synteny and collinearity. Nucleic Acids Res.

[70] Katoh K, Standley DM (2013). MAFFT multiple sequence alignment software version 7: improvements in performance and usability. Mol Biol Evol.

[71] Suyama M, Torrents D, Bork P (2006). PAL2NAL: robust conversion of protein sequence alignments into the corresponding codon alignments. Nucleic Acids Res.

[72] Stamatakis A (2014). RAxML version 8: a tool for phylogenetic analysis and post-analysis of large phylogenies. Bioinformatics (Oxford, England).

[73] Schumacher C, Krannich CT, Maletzki L, Köhl K, Kopka J, Sprenger H, Hincha DK, Seddig S, Peters R, Hamera S, Zuther E, Haas M, Horn R (2021). Unravelling differences in candidate genes for drought tolerance in potato (Solanum tuberosum l.) by use of new functional microsatellite markers. Genes.

[74] Savić J, Dragićević I, Pantelić D, Oljača J, Momćilović I (2012). Expression of small heat shock proteins and heat tolerance in potato (Solanum tuberosum L.). Arch Biol Sci.

[75] Mariot RF, de Oliveira LA, Voorhuijzen MM, Staats M, Hutten RCB, Van Dijk JP, Kok E, Frazzon J (2015). Selection of reference genes for transcriptional analysis of edible tubers of potato (Solanum tuberosum L.). PLOS ONE.

[76] Pfaffl MW (2001). A new mathematical model for relative quantification in real-time RT-PCR. Nucleic Acids Res.

