# Supplementary material for: Genome-wide identification and expression analysis of the SET domain-containing gene family in potato (Solanum tuberosum L.)
Source: BMC Genomics. 2024 May 3;25:442. doi: 10.1186/s12864-024-10367-2 (PMC11069243; doi:10.1186/s12864-024-10367-2)
Supplement: Supplementary file 2 — Supplementary Material 2. [file 12864_2024_10367_MOESM2_ESM.pdf]

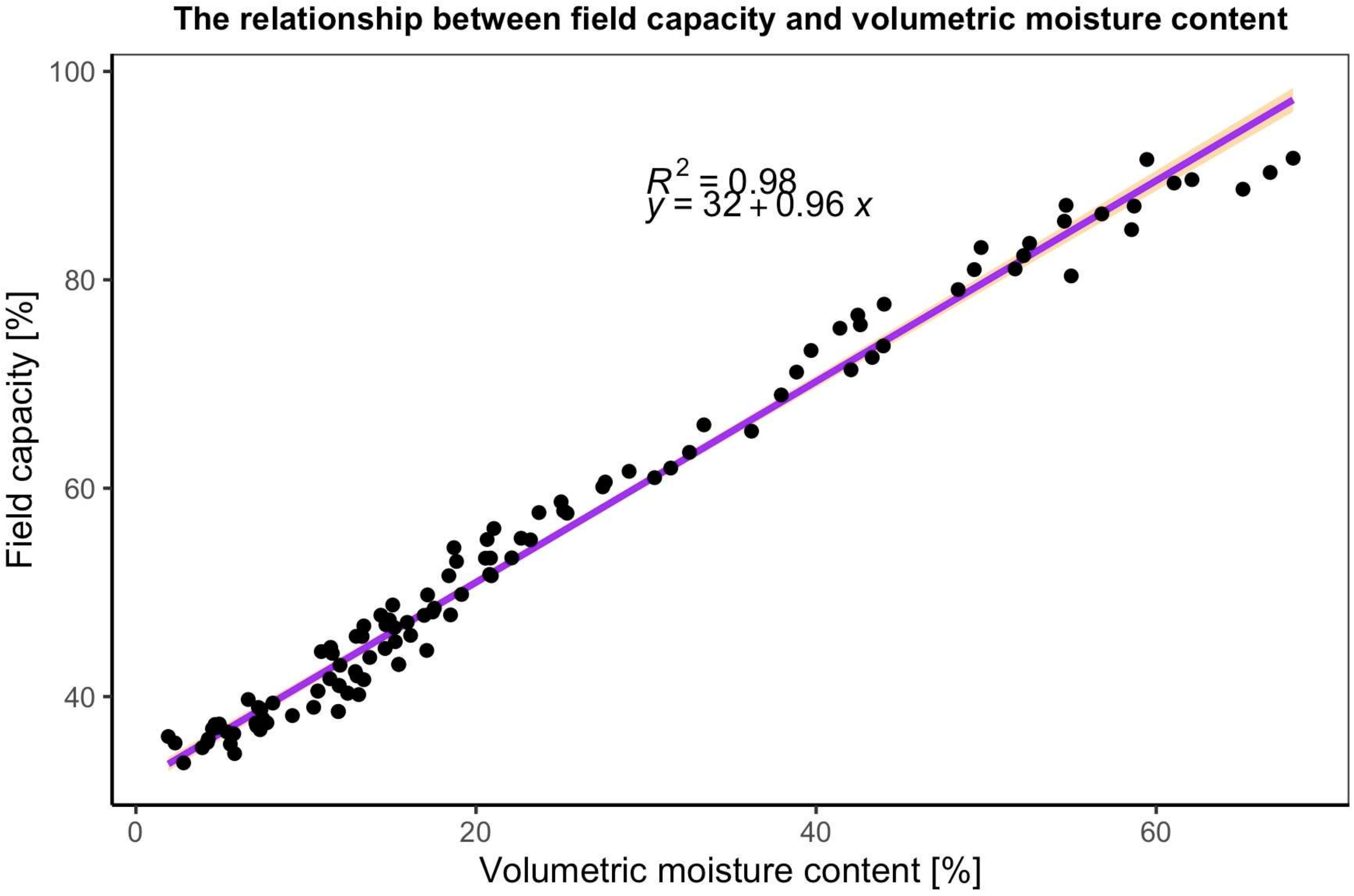

Figure S1: The linear relationship between field capacity and volumetric moisture content was deciphered to determine the amount of water that was added to the plants. The linear relationship between field capacity [%] and volumetric moisture content [%] is plotted in RStudio v.1.4.1717.

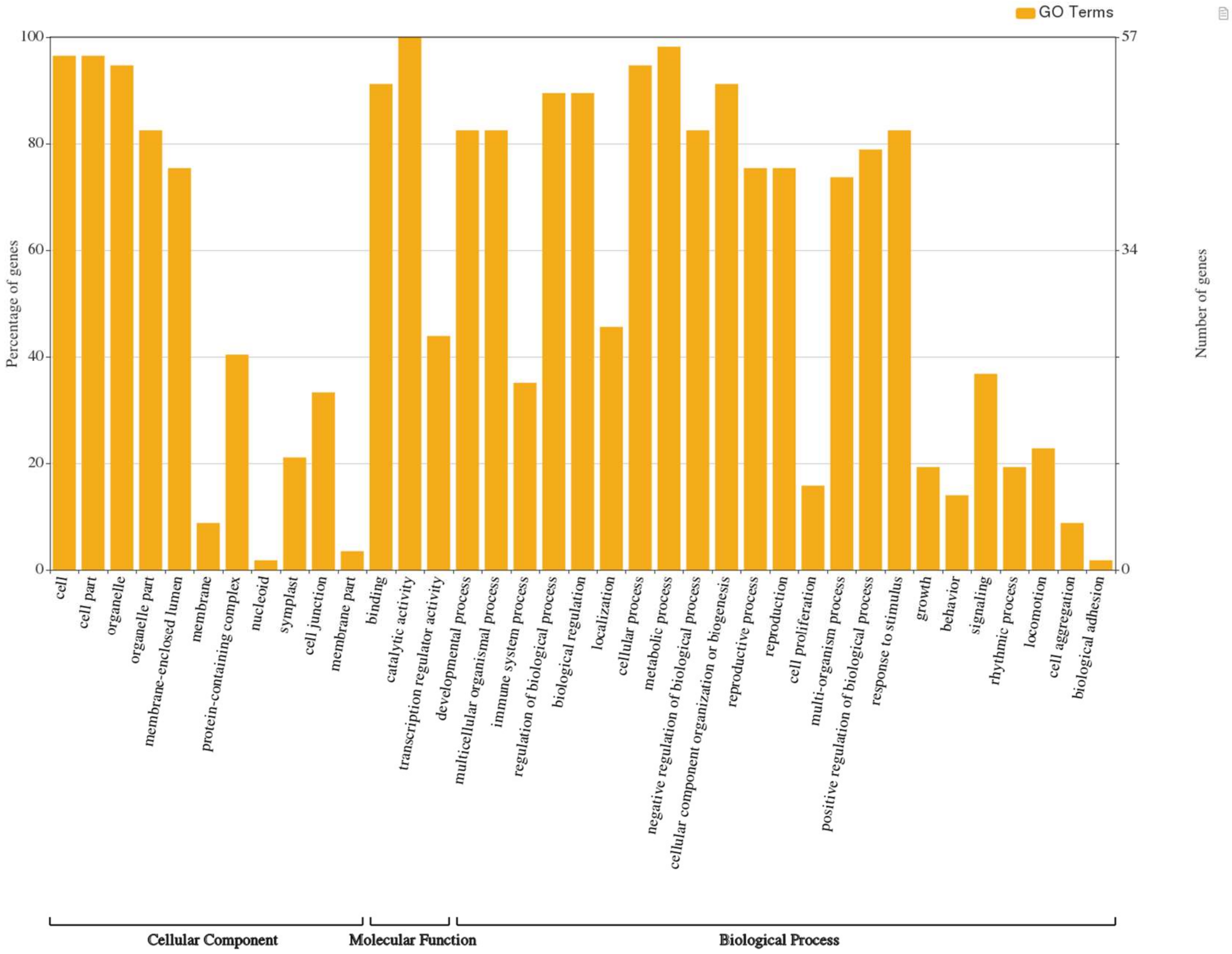

Figure S2: Overrepresented Gene Ontology terms identified in StSET genes using WEGO 2.0 [59].

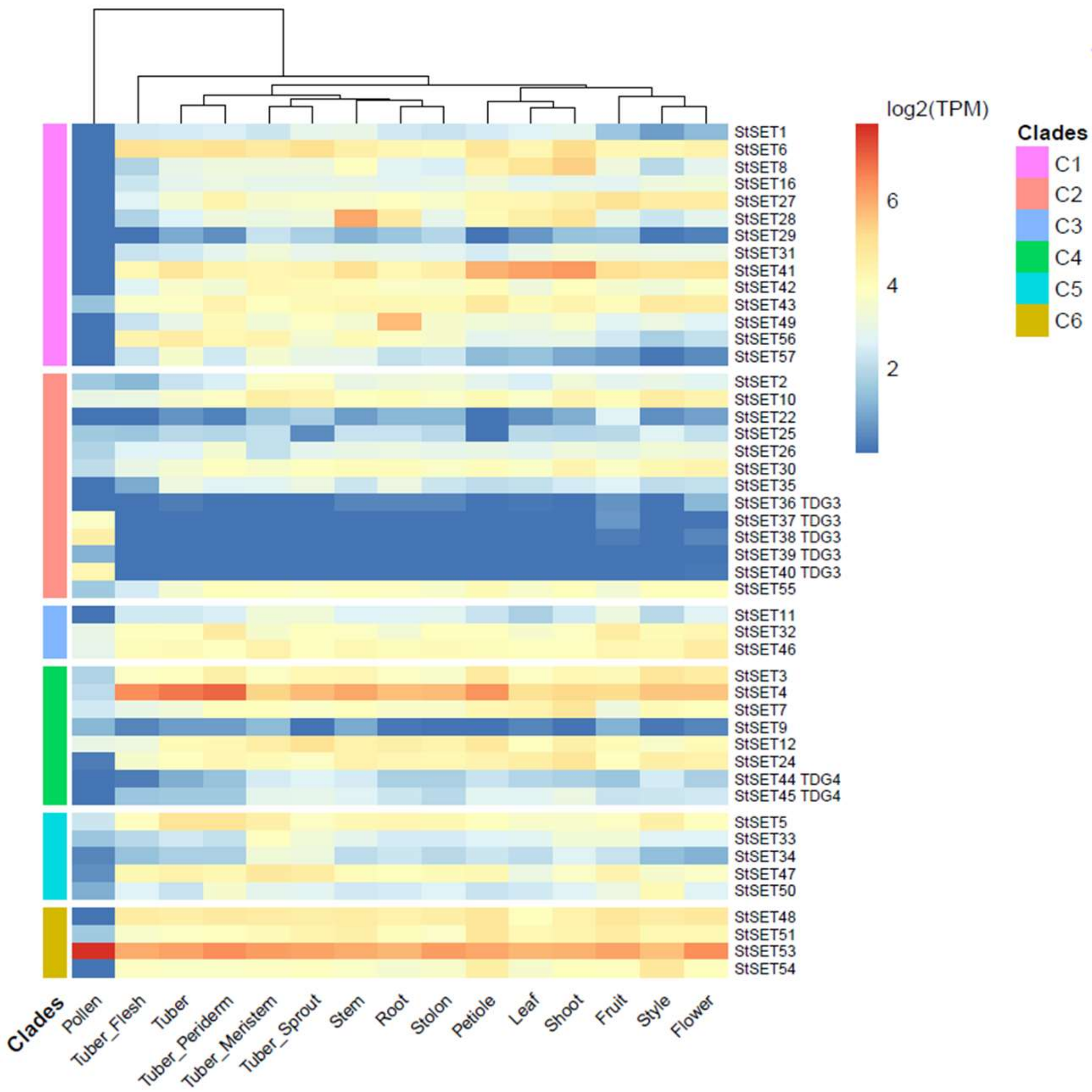

Figure S3: Global expression patterns of StSET genes in fifteen different tissues. Without clustering rows. Three genes, such as StSET37, StSET38, and StSET40, showed tissue-specific expression in pollen with an average Tau index of 0.9928. The expression values are log-transformed transcripts per million (TPM). The TPM values are retrieved from StCoExpNet [33].
